# Expanding the Anatomical Distribution of *PRRX1::KMT2D* Fusion Mesenchymal Neoplasms: A Rare Mediastinal Case Report

**DOI:** 10.1002/cnr2.70516

**Published:** 2026-03-08

**Authors:** Weixiang Zhong, Yu Deng, Ke Sun

**Affiliations:** ^1^ Department of Pathology, The First Affiliated Hospital Zhejiang University School of Medicine Hangzhou China

**Keywords:** case report, gene fusion, mediastinum, mesenchymal neoplasm, *PRRX1::KMT2D*

## Abstract

**Background:**

*PRRX1*‐rearranged mesenchymal neoplasms are rare soft tissue tumors with a predilection for the superficial subcutaneous tissue. The *PRRX1::KMT2D* fusion variant is exceptionally rare, with only three previously reported cases, all of which were located in the intermuscular regions. However, its occurrence in deep visceral sites has not been documented.

**Case:**

A 62‐year‐old woman was admitted after a routine physical examination revealed a space‐occupying lesion in the left thoracic cavity. Contrast‐enhanced CT showed a mixed‐density mass (10.4 × 8.1 × 3.8 cm) at the left cardiophrenic angle. The patient underwent complete thoracoscopic resection. Intraoperative frozen sections suggested a spindle cell tumor. Postoperative pathology, immunohistochemistry, and targeted RNA sequencing identified a *PRRX1::KMT2D* fusion mesenchymal neoplasm. At 18‐month follow‐up, no recurrence or progression was observed.

**Conclusion:**

This is the first reported case of a *PRRX1::KMT2D* fusion mesenchymal neoplasm arising in the mediastinum, which expands the anatomical spectrum of this emerging entity. Our findings underscore the importance of integrating morphological, immunohistochemical, and molecular approaches for accurate diagnosis, particularly in deep‐seated and unusual locations.

## 1 Introduction

In 2019, Lacambra et al. first described a group of mesenchymal neoplasms characterized by *PRRX1* rearrangements, predominantly involving *NCOA1* or *NCOA2* as fusion partners [1]. Subsequent studies expanded the molecular spectrum to include *KMT2D* as a rare but recurrent fusion partner [2, 3, 4]. To date, only three genetically confirmed *PRRX1::KMT2D* fusion tumors have been reported, all situated in the intermuscular septa of the chest wall, knee, and calf muscles. Histologically, these neoplasms are characterized by hypocellular, bland spindle‐to‐stellate cells embedded within a vascularized myxocollagenous stroma [2, 3, 4].

Despite increasing recognition, the anatomical distribution and histological spectrum of *PRRX1::KMT2D* fusion neoplasms remain incompletely characterized. No case has been documented in visceral or intrathoracic locations prior to this report, and their distinction from histological mimics, such as *NTRK*‐rearranged tumors, *KMT2A*‐rearranged sarcomas, and *RB1*‐deleted neoplasms, remains diagnostically challenging [5, 6, 7, 8].

Herein, we present the first documented case of a *PRRX1::KMT2D* fusion mesenchymal neoplasm arising in the mediastinum. This unique presentation expands the anatomical repertoire of this emerging entity, highlights potential diagnostic pitfalls (particularly the interpretation of focal S100/SOX10/NTRK expression), and underscores the utility of targeted RNA sequencing in classifying unselected spindle cell neoplasms. By reporting this case, we aim to increase diagnostic awareness and support the inclusion of *PRRX1::KMT2D* fusion neoplasms in the differential diagnosis of deep‐seated myxoid spindle cell neoplasms.

## 2 Case Report

**FIGURE 1:**
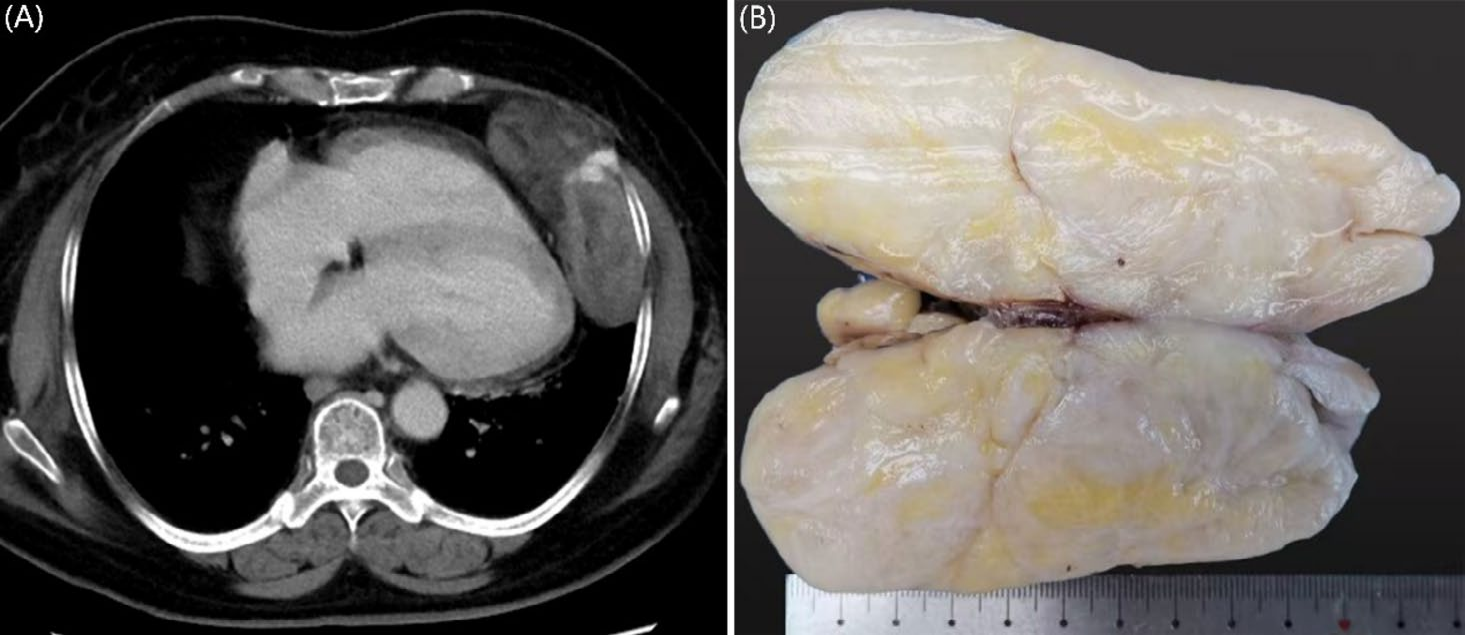
(A) CT image showing a heterogeneous 10.4 cm mass in the left cardiophrenic angle. (B) Gross specimen demonstrating a well‐defined, multinodular, oval mass without apparent adhesion to surrounding soft tissue. The cut surface revealed firm to translucent, with focal gelatinous areas and a pale yellow appearance.

A 62‐year‐old woman was incidentally found to have a thoracic mass during a routine physical examination in June 2024. She had a history of mild coronary artery stenosis and reported no chest pain, dyspnea, cough, or constitutional symptoms. On June 26, the patient was admitted to the Department of Thoracic Surgery at the First Affiliated Hospital of Zhejiang University School of Medicine for further evaluation and management of the condition. Contrast‐enhanced chest computed tomography (CT) revealed a heterogeneous mass measuring 10.4 cm in size located in the left cardiophrenic angle. The lesion demonstrated several characteristic features: (1) linear and patchy areas of fat attenuation, (2) heterogeneous contrast enhancement patterns ranging from focal intensity to mild, and (3) localized posterior pleural thickening. Mild fatty liver and calcification of the aortic and coronary vessel walls were also noted. No significant lymphadenopathy or pleural effusion was observed (Figure 1A).

**FIGURE 2:**
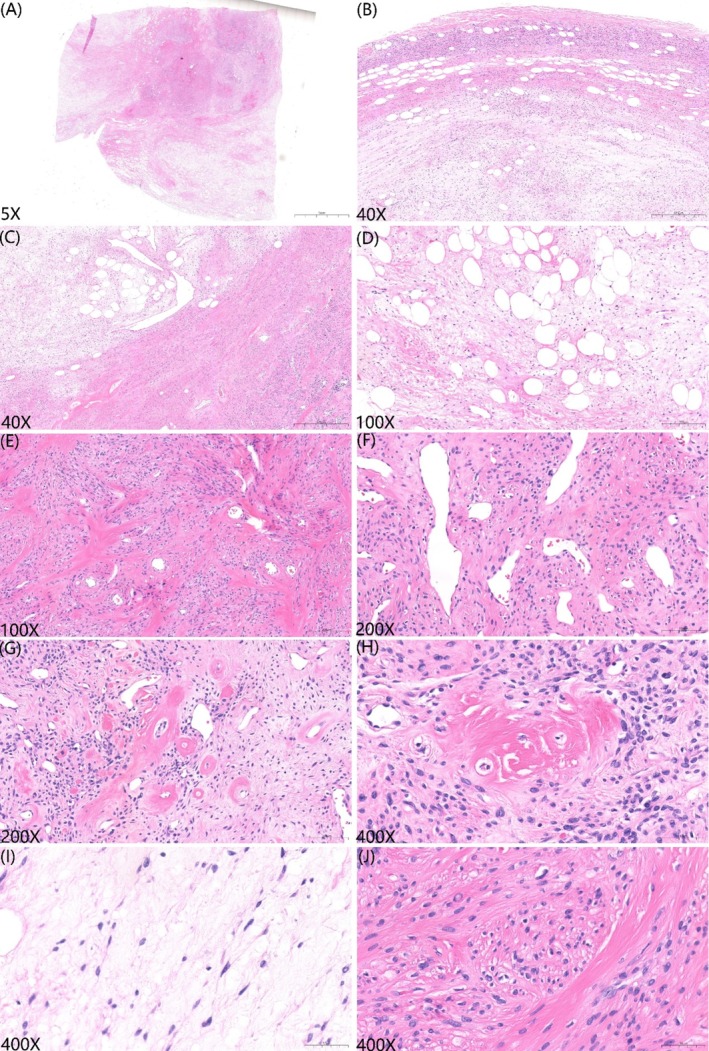
Histopathological features of the specimen. (A, B) The tumor was well‐circumscribed with a thin fibrous capsule in certain regions. (C, D) The tumor consisted of myxoid and collagenized areas interspersed with mature adipose tissue. (E) Collagenized areas showed increased cellularity, arranged in loose fascicles and cords. (F, G) Irregularly dilated gaping/staghorn‐like blood vessels with prominent perivascular hyalinization. (H) Distinctive rosette‐like structures composed of increased neoplastic cells surrounding hyalinized vascular. (I, J) Tumor cells are sparse in the myxoid areas and consist of stellate‐to‐spindle‐shaped cells with small ovoid to elongated nuclei. In the collagenized areas, the cell density increased, with monomorphic ovoid to spindle‐shaped cells.

The initial clinical impression suggested a benign lesion. After comprehensive preoperative evaluation ruled out surgical contraindications, the patient underwent complete thoracoscopic resection of the mediastinal tumor under general anesthesia on July 5, 2024. Histopathological examination confirmed negative margins. Gross examination revealed a well‐circumscribed, multinodular ovoid mass measuring 10.4 cm in greatest diameter without adhesion to adjacent soft tissues. The cut surface was variegated and composed of firm, translucent parenchyma interspersed with gelatinous areas and pale yellow discoloration (Figure 1B). Microscopic examination revealed well‐demarcated margins with subtly lobulated architecture (Figure 2A,B), featuring alternating myxoid and collagenized areas interspersed with mature adipose tissue (Figure 2C,D). The collagenous components included centrally dense coarse collagen bundles encircled by prominent irregular crescentic or “staghorn”‐patterned vascular channels displaying focal perivascular hyalinization (Figure 2E–G). At higher magnification, neoplastic cells within the myxoid regions were predominantly stellate‐shaped and presented faintly eosinophilic to clear cytoplasm and small ovoid‐to‐elongated nuclei harboring finely dispersed granular chromatin with inconspicuous nucleoli. In contrast, the collagenous zones predominantly consisted of ovoid‐spindle cells exhibiting eosinophilic cytoplasm and small ovoid‐to‐ovoid spindle nuclei, frequently demonstrating perinuclear halos and mild cytological atypia. However, mitotic activity and necrosis were absent (Figure 2I,J). Of particular diagnostic significance were the distinctive rosette‐like formations comprising neoplastic cells radially oriented around hyalinized vascular cores (Figure 2H).

**FIGURE 3:**
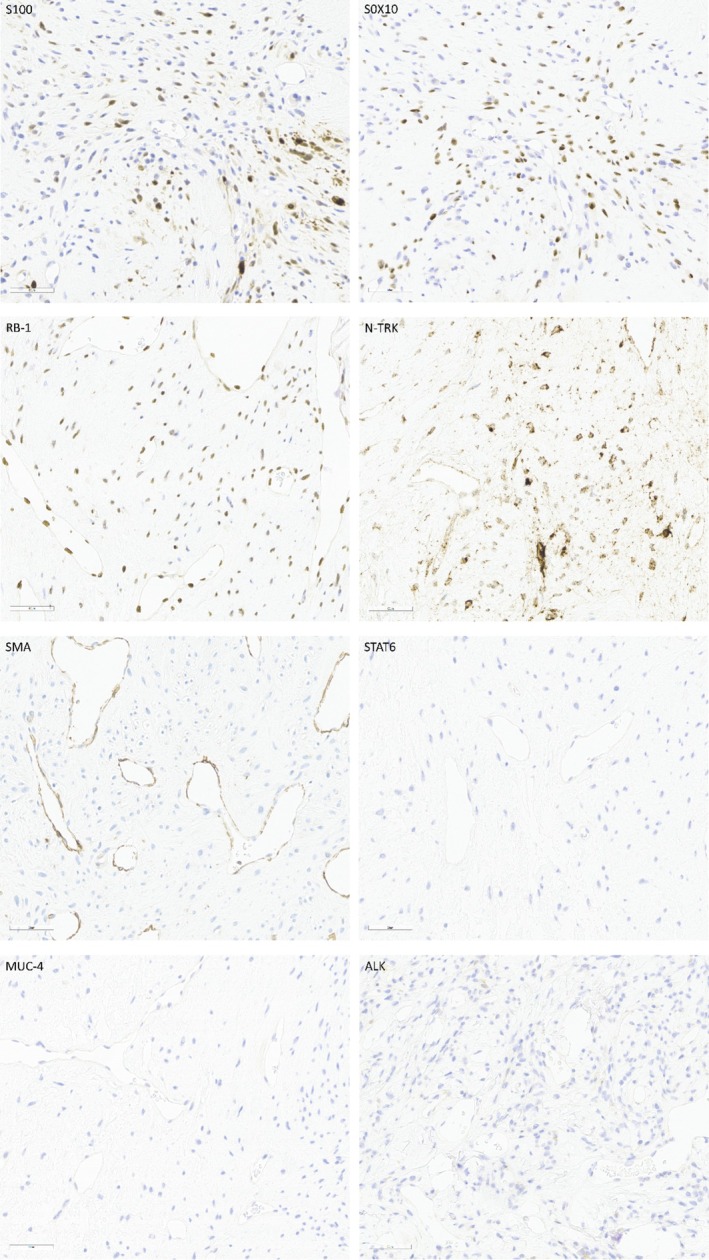
Immunohistochemical staining revealed that tumor cells focally co‐expressed S100 and SOX10, were positive for NTRK, and retained RB1 expression, while remaining consistently negative for epithelial markers, SMA, STAT6, MUC4, and ALK.

**FIGURE 4:**
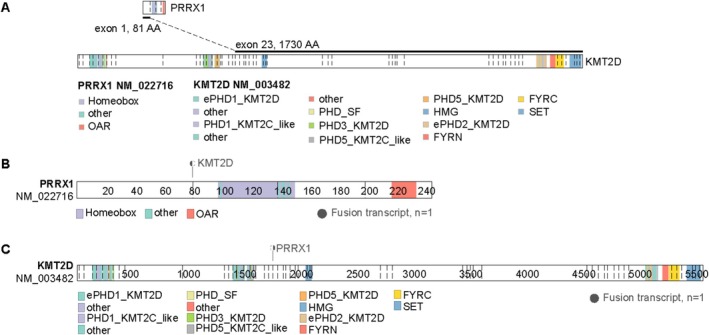
Targeted RNA next‐generation sequencing identified fusion transcripts of *PRRX1* (NM022716) (exon 1) and *KMT2D* (NM003482) (exon 23). (A) Fusion occurs between exon 1 of *PRRX1* and exon 23 of *KMT2D*, with the fusion site denoted by a bold black line and distinct colors representing the predicted domains within the protein. (B) Fusion of *PRRX1* with *KMT2D* results in the loss of domains, such as Homeobox and OAR from *PRRX1*. (C) Similarly, fusion leads to the loss of domains, such as ePHD1_*KMT2D*, from *KMT2D;* whereas domains, such as HMG, remain preserved.

Immunohistochemical analysis revealed that tumor cells focally co‐expressing S100 and SOX10 were positive for NTRK and retained RB1 expression, while remaining consistently negative for epithelial (CKpan, EMA), myogenic (SMA), and other mesenchymal markers (CD34, STAT6, MUC4, DOG‐1, and CD117), with additional negativity for ALK. The proliferative activity, as assessed by the Ki‐67 labeling index, was low (2%) in areas with the highest degree of proliferation (Figure 3). Molecular analysis via targeted RNA sequencing (TruSight RNA Fusion Panel; Illumina, San Diego, CA, USA) identified a novel in‐frame fusion transcript between exon 1 of *PRRX1* (NM_022716) and exon 23 of *KMT2D* (NM_003482) (Figure 4).

Based on comprehensive histopathological evaluation, immunohistochemical profiling, and molecular genetic analysis, the tumor was definitively diagnosed as a *PRRX1::KMT2D* rearranged mesenchymal neoplasm. The patient underwent complete surgical resection with negative margins. No adjuvant therapy was administered. Follow‐up examinations were performed every 6 months after surgery. A chest radiograph performed in January 2026 showed no evidence of tumor recurrence or metastasis.

## 3 Discussion

This case provides several key insights into the understanding of *PRRX1::KMT2D* fusion mesenchymal neoplasms. First, it documents the first mediastinal occurrence, thereby expanding the anatomical spectrum of this rare entity. Second, it confirms that deep‐seated *PRRX1::KMT2D* tumors can follow an indolent course similar to their superficial counterparts, in contrast to aggressive *KMT2A*‐rearranged sarcomas [2, 7]. Third, it highlights a diagnostic pitfall: focal S100/SOX10 co‐expression and NTRK positivity may mimic neural or *NTRK*‐rearranged tumors, but the absence of CD34 co‐expression and infiltrative growth, together with molecular confirmation, permit accurate classification [9]. Fourth, it underscores the utility of RNA sequencing in the evaluation of unclassified spindle cell neoplasms, particularly when immunohistochemistry yields ambiguous results.

To date, including the present case, 29 *PRRX1*‐rearranged mesenchymal neoplasms have been reported in the literature [1, 2, 3, 4, 5, 6, 9, 10, 11, 12]. Molecular analysis revealed *NCOA1* as the predominant fusion partner (83%, 24/29), followed by *KMT2D* (14%, 4/29) and *NCOA2* (3%, 1/29). These tumors occurred predominantly in adults (median age: 40 years; range 20–76 years), with tumor sizes ranging from 2.0 to 15.8 cm (median: 4.0 cm), and a slight female predominance (F:M = 18:11). Anatomically, most tumors (83%, 24/29) arose in superficial subcutaneous tissue, whereas 17% (5/29) originated in deep soft tissues (Table 1). Notably, the type of fusion partner appears to correlate with anatomical distribution: *PRRX1::NCOAx* tumors are almost exclusively subcutaneous (24/25), with only a single reported intermuscular case [3]. In contrast, all *PRRX1::KMT2D*‐rearranged tumors (4/4) presented deep‐seated origins: one mediastinal (present case) and three intermuscular (prior reports) [2, 3, 4]. Our report of a 10.4 cm mediastinal *PRRX1::KMT2D* neoplasm in a 62‐year‐old woman represents the first well‐documented occurrence outside subcutaneous or intermuscular sites.

**TABLE 1:** Summary of clinicopathological features of PRRX1‐rearranged mesenchymal tumors.

Case	Authors	Age/sex	Location	Size (cm)	S100	SOX10	Gene fusion	Follow‐up (months)
1	Lacambra et al.	55/F	Thigh/S	4	P(weak)	NA	PRRX1ex1::NCOA1ex13	ANED (24)
2		33/M	Neck/S	14	N	NA	PRRX1ex1::NCOA1ex13	ANED (6–18)
3		43/F	Neck/S	3	N	N	PRRX1ex1::NCOA1ex13	ANED (6–18)
4		21/F	Groin/S	2	N	NA	PRRX1ex1::NCOA2ex15	ANED (6–18)
5	Puls et al.	40/M	Knee/intramuscular	13	N	NA	PRRX1ex1::KMT2D ex22	ANED (24)
6	Dermawan et al.	49/M	Abdominal wall/S	4	N	NA	PRRX1ex1::NCOA1ex13	ANED (2)
7		43/M	Axilla/S	5.5	N	NA	PRRX1ex1::NCOA1ex13	ANED (3)
8		34/F	Shoulder/S	N/A	N	NA	PRRX1ex1::NCOA1ex13	N/A
9		41/F	Abdominal wall/S	4	N	N	PRRX1ex1::NCOA1ex13	N/A
10		76/F	Abdominal wall/S	2.6	N	N	PRRX1ex1::NCOA1ex13	ANED (2)
11		20/M	Hip/S	5	N	NA	PRRX1ex1::NCOA1ex13	ANED (1.6)
12	Chen et al.	23/M	Scalp/S	2.9	N	N	PRRX1ex1::NCOA1ex13	ANED (26)
13		46/M	Groin/S	5	N	NA	PRRX1ex1::NCOA1ex13	ANED (7)
14	Cloutier et al.	23/M	Shoulder/S	2.5	P(focal)	N	PRRX1ex1::NCOA1ex13	N/A
15	Warmke et al.	46/F	Neck/S	2.2	P(focal)	P(focal)	PRRX1ex1::NCOA1ex13	ANED (4)
16		36/M	Neck/S	3.4	P(focal)	P(focal)	PRRX1ex1::NCOA1ex13	ANED (5)
17		65/F	Chest wall/intramuscular	4	P(focal)	P(focal)	PRRX1ex1::KMT2Dex25–27	ANED (12)
18		37/F	Flank/S	9.5	N	N	PRRX1ex1::NCOA1ex13	N/A
19		29/M	Forehead/frontal muscle	2.5	N	N	PRRX1ex1::NCOA1ex13	N/A
20		56/F	Back/S	3	P(focal)	N	PRRX1ex1::NCOA1ex13	ANED (16)
21	Cheng et al.	26/F	Left thigh/S	4	P(focal)	P(focal)	PRRX1ex1:: NCOA1ex15	ANED (13)
22	Cordier et al.	50/M	Shoulder/S	2.7	P	N	PRRX1ex1::NCOA1ex13	N/A
23		40/F	Shoulder/S	NA	N	N	PRRX1ex1::NCOA1ex13	N/A
24	Grosse et al.	22/F	Supraclavicular region/S	3	N	N	PRRX1ex1::NCOA1ex13	ANED (12)
25	Rongfen et al.	40/F	Right calf/intramuscular	15.8	P(focal)	P(focal)	PRRX1ex1::KMT2Dex22	ANED (1)
26		28/F	Right thigh/S	4	N	N	PRRX1ex1::NCOA1ex13	ANED (12)
27		26/F	Left thigh/S	4	N	N	PRRX1ex1:: NCOA1ex15	ANED (14)
28		42/F	Flanks/S	5.5	P(focal)	P(focal)	PRRX1ex1:: NCOA1ex15	ANED (3)
29	Present case	62/F	Mediastinum	10.4	P(focal)	P(focal)	PRRX1ex1::KMT2Dex23	ANED (11)

Abbreviations: ANED, alive with no evidence of disease; F, female; M, male; N, negative; NA, not available; NED, no evidence of recurrence; P, positive; S, subcutis.

Histopathologically, all *PRRX1*‐rearranged neoplasms share characteristic features, including a well‐circumscribed multinodular architecture, bland spindle cells in myxocollagenous stroma with “staghorn” vasculature, and variable additional findings (adipocytes, mast cells, and collagen rosettes). Immunohistochemically, the tumors were consistently negative for SMA, desmin, MUC4, STAT6, CD34, and EMA. Focal S100 (38%, 11/29) and SOX10 (25%, 7/20) reactivity [1, 2, 3, 4, 5, 6, 9, 10, 11, 12] suggest partial neuroectodermal differentiation [3]. This immunoprofile, particularly the concurrent S100/SOX10 expression, may mimic neural tumors, posing a potential diagnostic pitfall [3, 9]. Further challenges include RB1 deletion (FISH) and NTRK/ALK expression without molecular alterations [3, 10, 12].

The biological implications of *PRRX1* and *KMT2D* alterations may underlie clinicopathological observations. *PRRX1* (1q24.2), a paired‐related homeobox transcription factor, acts as a transcriptional coactivator that regulates morphogenesis [13]. These rearrangements are associated with therapy‐related leukemia, myelodysplastic syndromes, and musculoskeletal developmental disorders [3]. *KMT2D* (12q13.12, also designated *MLL2*) encodes a histone methyltransferase critical for differentiation and tumor suppression with mutations that are recurrent in lymphomas, carcinomas, and sarcomas [14, 15, 16]. The pathogenic role of *PRRX1::KMT2D* fusion in mesenchymal neoplasms requires further investigation.

Cumulative evidence supports an indolent course for *PRRX1*‐rearranged tumors. Accurate diagnosis is critical for distinguishing these lesions from histological mimics with malignant potential. Clinically, the differential diagnoses include thymoma, schwannoma, and teratoma. Definitive differentiation can be achieved through pathological examination. Pathological morphology overlaps with various tumors, including low‐grade fibromyxoid sarcoma (LGFMS), sclerosing epithelioid fibrosarcoma (SEF), angiofibroma, ossifying fibromyxoid tumor (OFMT), fibrosarcoma, and *RB1*‐deficient tumors (such as spindle cell lipoma). It is especially important to distinguish it from KMT2A‐rearranged sarcoma, which typically exhibits infiltrative growth, high‐grade morphology, and aggressive behavior [7, 8]. Although focal S100/NTRK immunoreactivity may suggest an *NTRK*‐rearranged tumor, the latter usually co‐expresses CD34 and is negative for SOX10 (Table 2 for a comprehensive comparison).

**TABLE 2:** Pathological differential diagnosis of PRRX1‐rearranged mesenchymal neoplasms.

Tumor type	Key diagnostic features	Molecular characteristics
LGFMS	“Abrupt” myxoid nodules, curvilinear blood vessels, MUC4 positive	FUS/EWSR1::CREB3L1/L2/L3/CREM fusion
SEF	High‐grade nuclear atypia, MUC4 positive	FUS/EWSR1::CREB3L1/L2/L3/CREM fusion
SFT	CD34/STAT6 positive, alternating hypercellular and hypocellular areas	NAB2‐STAT6 fusion
RB1‐deficient tumor family	Loss of RB1 protein, CD34/ER positive (cellular angiofibroma/mammary‐type myofibroblastoma) RB1 deletion	RB1 deletion
Soft tissue angiofibroma	EMA positive, variable vascular patterns	NCOA2 fusion (AHRR/GTF2I)
KMT2A‐YAP1 fusion sarcoma	High‐grade morphology, infiltrative growth, aggressive behavior	KMT2A fusion
NTRK‐rearranged tumors	Coexpression of CD34/S100, SOX10 negative, infiltrative growth	NTRK fusion
Nerve sheath tumors	Diffuse strong S100/SOX10 positivity (neurofibroma), EMA/claudin‐1 positive (perineurioma)	
Desmoid fibromatosis	Nuclear β‐catenin positivity	CTNNB1 mutation
OFMT	Desmin S100 CD10 and TFE3 positive	PHF1 rearrangement
Nasopharyngeal angiofibroma	Rare nasal tumor predominantly affecting adolescent males	CTNNB1 mutation

From a clinical perspective, recognition of this entity is critical to avoid misdiagnosis as a sarcoma and prevent overtreatment. Current evidence supports the classification of these tumors as *PRRX1*‐rearranged mesenchymal tumors rather than fibroblastic tumors [3]. We therefore recommend that *PRRX1::KMT2D* fusion neoplasms be included in the differential diagnosis of mediastinal myxoid spindle cell tumors. Definitive diagnosis relies on a systematic approach, including (1) identification of characteristic morphological features, (2) immunohistochemistry suggesting possible partial neural or neuroectodermal differentiation to exclude aggressive tissue mimics, and (3) molecular confirmation of pathogenic fusions, an algorithmic strategy particularly crucial for accurately diagnosing unclassifiable lesions encountered in limited biopsy samples.

## 4 Conclusion

In conclusion, we report the first mediastinal *PRRX1::KMT2D* fusion mesenchymal neoplasm, expanding the anatomical and morphological spectrum of this emerging entity. Our findings emphasize the importance of a multimodal diagnostic approach integrating morphological, immunohistochemical, and targeted RNA sequencing analyses. Furthermore, this case supports the indolent nature of this neoplasm. Long‐term surveillance and further case accumulation are warranted to delineate its biological and clinical characterization.

## Author Contributions

All authors have made substantial intellectual contributions to this work. Weixiang Zhong was responsible for the conception of the study, data acquisition and analysis, and drafting the initial manuscript. Yu Deng participated in data interpretation and critically revised the manuscript for important intellectual content. Ke Sun supervised the project, provided final approval of the version to be published, and is the corresponding author. All authors read and approved the final manuscript.

## Funding

The authors have nothing to report.

## Ethics Statement

This study received ethical approval and consent for participation from the Clinical Research Ethics Board of the First Affiliated Hospital, Zhejiang University School of Medicine. The images used in this study did not contain any patient records or other identifiable information.

## Consent

The authors have nothing to report.

## Conflicts of Interest

The authors declare no conflicts of interest.

## Data Availability

The data that support the findings of this study are available on request from the corresponding author. The data are not publicly available due to privacy or ethical restrictions.
